# Brief international cognitive assessment for MS (BICAMS) and NEDA maintenance in MS patients: A 2‐year follow‐up longitudinal study

**DOI:** 10.1111/ene.70007

**Published:** 2024-12-21

**Authors:** E. Leveraro, M. Cellerino, C. Lapucci, M. Dighero, L. Nasone, T. Sirito, D. Boccia, N. Cavalli, G. Bavestrello, A. Uccelli, G. Boffa, M. Inglese

**Affiliations:** ^1^ Department of Neuroscience, Rehabilitation, Ophthalmology, Genetics, and Mother‐Child Health (DINOGMI) University of Genoa Genoa Italy; ^2^ IRCCS Ospedale Policlinico San Martino Genoa Italy

**Keywords:** BICAMS, cognitive impairment, high‐efficacy therapy, multiple sclerosis

## Abstract

**Background:**

The Brief International Cognitive Assessment for Multiple Sclerosis (BICAMS) has been validated in many cross‐sectional studies. However, longitudinal data on BICAMS subset trajectories and their correlation with disease activity during follow‐up are scarce.

**Objectives:**

We aimed to (i) assess BICAMS changes in MS patients initiating high‐efficacy disease‐modifying‐treatments (DMTs), (ii) compare these changes based on maintenance of “no‐evidence‐of‐disease‐activity” (NEDA‐3) status over 24 months, and (iii) determine baseline clinical parameters predictive of cognitive changes.

**Methods:**

We enrolled 101 MS patients (mean age:40,45 ± 11; Relapsing–Remitting‐MS:81%) initiating highly‐effective‐DMTs. Patients underwent Expanded Disability Status Scale (EDSS), BICAMS, and Hospital Anxiety and Depression Scale (HADS), at baseline and after 24 months. Regression‐based change index (RB‐CI) had been used for cognitive change evaluation over follow‐up.

**Results:**

During follow‐up, 78 (77.3%) patients maintained NEDA‐3 status. Considering a 90% of confidence levels for RB‐CI, 12 (11.9%) improved at SDMT, 13 (12.9%) at CVLT‐II and 13 (12.9%) at BVMT‐R; while 7 (6.9%) were classified as worsened at SDMT, 11 (10.9%) at CVLT‐II and 8 (7.9%) at BVMT‐R. SDMT scores significantly improved at follow‐up for the entire group (*p* = 0.003) and in patients maintaining NEDA‐3 (*p* < 0.001). The multivariable regression model assessing the SDMT improvement (*n* = 12; *z* = 1.65), was significant and explained 21% of the variance (*p* = 0.038; Nagelkerke *R* ^2^ = 0.212). Lower EDSS proved to be an independent predictor of SDMT reliable improvement (*p* = 0.027) in our sample.

**Conclusions:**

Our findings showed that early disease activity control—especially in patients with low baseline disability—may yield significant benefits even in terms of cognitive performance.

## INTRODUCTION

Multiple Sclerosis (MS) is a chronic immune‐mediated disease affecting the Central Nervous System (CNS), characterized by inflammatory, demyelinating, and degenerative processes [1]. Cognitive impairment, a prevalent and debilitating manifestation in MS, impacts processing speed, memory, and executive function [2, 3, 4].

Currently, the most widely used outcome measure in MS monitoring is “no evidence of disease activity” (NEDA‐3) composite measure, which consists of no relapses and Expanded Disability Status Scale (EDSS) progression, and no radiological activity (defined as no new and/or enlarging T2 lesions and gadolinium‐enhancing lesions) [5]. Patients who maintain NEDA‐3 status are generally considered stable but recent findings have shown that cognitive deterioration might still occur in these individuals [6].

Consensus opinion considers the Symbol Digit Modalities Test (SDMT) the best measure of cognitive processing speed (CPS), a core cognitive symptom in MS [7]. Recent research underscores SDMT's reliability and validity, highlighting it as the sole CPS test with clinically meaningful score changes [7]. The Brief International Cognitive Assessment for Multiple Sclerosis (BICAMS)—which includes SDMT—is a commonly used neuropsychological test battery in MS [8]; however, only a few studies have presented results from longitudinal assessments of BICAMS subtests over longer intervals, especially in the context of treatment response [9, 10, 11]. To prevent inadequate interpretation of efficacy of both rehabilitative and pharmacological interventions, recently, Portaccio et al [12, 13] provided normative data for reliable cognitive change index (RCI) and regression‐based change index (RB‐CI) for MACFIMS subtests (which includes the SDMT, CVLT‐II, and BVMT‐R) providing new thresholds for interpreting change in each test. Since early high‐efficacy therapy initiation has been shown to prevent disability accrual over time [14, 15, 16]. we hypothesized that efficient treatment‐induced disease control could also prevent cognitive worsening. Moreover, recent reports highlight the possibility of cognitive improvement in MS patients undergoing high‐efficacy treatment suggesting a possible cognitive improving effect of DMTs [17]. The present study aimed to: (i) assess BICAMS subtest changes over 24 months in MS patients beginning highly effective disease‐modifying treatments (DMTs); (ii) compare cognitive trajectories between patients maintaining and those losing NEDA‐3 status over follow‐up; (iii) identify baseline clinical parameters predictive of statistically‐meaningful cognitive changes over time.

## METHODS

### Subjects

In total, 101 MS patients were prospectively enrolled in this longitudinal cohort study. Inclusion criteria were: (i) age 18–65 years; (ii) MS diagnosis according to 2017 McDonald's criteria [1]; (iii) commencement of a high‐efficacy therapy (cladribine, fingolimod, natalizumab or anti‐CD20 drugs) [14, 18]‐ within 3 months of study onset. Exclusion criteria were: (i) neurological disorders other than MS that might influence cognitive functioning (e.g., dementia or brain injury); (ii) concomitant psychiatric or personality disorders diagnosis that could influence cognitive performance (e.g., dissociative disorders, schizophrenia, or psychotic symptoms). Among all the patients who commenced high‐efficacy therapy, only those who consented to participate in all assessments at baseline and after 24 months, as well as signed informed consent for the use of their medical history for publication, were included in the study. High‐efficacy therapy was prescribed and administered by the treating physician to relapsing and progressive forms of MS following a tailored approach evaluating the profile risk of each patient, according to regulatory policies. All procedures were approved by the institutional review board.

### Clinical and mood evaluation

All subjects underwent standard neurological evaluation, including EDSS scoring [19] at baseline and after 24 months. Information regarding the occurrence of clinical relapses, radiological activity, and disability progression during follow‐up were collected. Information regarding the number of new T2/gadolinium‐enhancing lesions occurring during 12 months before enrolment was also collected [20].

Symptoms of anxiety and depression were assessed at baseline by the Hospital Anxiety and Depression Scale (HADS), a brief self‐reporting two‐dimensional questionnaire which is demonstrated to have a high sensitivity and specificity in MS evaluation [21].

### Cognitive evaluation

The Italian version of the BICAMS [8], was administered by a trained neuropsychologist to evaluate cognitive functions at baseline and follow‐up. The administrated battery included the oral version of the Symbol Digit Modalities Test—SDMT(form 1 and 2) [22], the California Verbal Learning Test, 2ndEd. —CVLT‐II (list A and list B) [23] and the Brief Visuospatial Memory Test Revised—BVMT‐R (Form 1 and form 2) [24]. Due to a potential learning effect, all test stimuli were different at baseline and follow‐up sessions, performing them in a randomized order to minimize practice effects when repeated [25, 26, 27].

The SDMT presents a series of nine symbols, each paired with a number in a legend at the top of a standard sheet of paper. Subjects are asked to verbally respond with the paired digits as quickly as possible when presented with a pseudo‐random sequence of symbols. The outcome measure of the SDMT is the number of correct responses in 90 seconds.

In the CVLT‐II, the examiner reads a list of 16 words. Subjects must recall the word list in the order they wish. This is repeated 5 times. The measured outcome is the total number of words recalled over the five trials.

The BVMT‐R measures the visuospatial learning and memory. Subjects are given 10s to memorize six abstract figures in a 2 × 3 grid; after this time, they are asked to remember and reproduce the stimuli on a sheet of paper. This is repeated 3 times. Each drawing is assigned a score of 0, 1, or 2 based on criteria of accuracy and positioning of the six figures. The dependent variable is the total recall score across the three trials.

The failure on each BICAMS subtest was defined based on Italian normative data [8] (T‐score ≤35). We interpreted the change in cognitive assessment from baseline to follow‐up using the latest normative data for both RCI and RB‐CI of SDMT, CVLT‐II, and BVMT‐R [13]. The reliable change index (RCI) uses test–retest reliability and score variability to establish a confidence interval within which a retest score is expected to fall. Consequently, if a retest score falls outside this interval, it likely indicates a genuine change. In contrast, the regression‐based change index (RB‐CI) methodology is designed to evaluate a predicted score for each individual based on age, sex, education, and the initial test score. Each follow‐up scaled score was then compared to the predicted scale score. Changes at each subtest were deemed significant by applying the 80% (*z* = 1.25) and 90% (*z* = 1.65) confidence interval.

### Statistical analyses

Statistical analyses were performed using SPSS version 23 (IBM Corp., Armonk, NY). Descriptive results included mean with standard deviation (SD), median with range, or number with percentage. Longitudinal cognitive performance changes on T scores were assessed using repeated‐measures analysis of covariance (ANCOVA). Binary logistic multivariable regression models explored associations between baseline demographic and disease‐related factors (disease duration, baseline EDSS, total HADS score, baseline MRI activity, and previous treatments) with BICAMS subtest worsening and improvement over follow‐up. Since the authors [13] reported that regression‐based methods are superior and preferable to RCIs, particularly for the assessment of improvement, in multivariate logistic regression analyses we applied RB‐CI with a confidence level of 90% (*z* = 1,65). All *p*‐values were two‐sided and considered statistically significant when *p* ⩽ 0.05.

## RESULTS

Demographic and clinical characteristics are reported in Table 1. At baseline, 17.8% of our sample performed below the cut‐off in at least one BICAMS subtest (T‐score ≤35).

**TABLE 1:** Clinical and demographic characteristics.

Total number of patients	101
Age, mean (SD)	40,45 (±11)
Female, number (%)	62 (61.4%)
Phenotype, number (%)	
RRMS	82 (81.2%)
PPMS	12 (11.9%)
SPMS	7 (6.9%)
Disease duration, mean (SD), years	9.06 (±10)
Treatment at baseline number (%)	
Ocrelizumab or Rituximab (anti‐CD20)	65 (64.3%)
Fingolimod	19 (18.8%)
Cladribine	13 (12.9%)
Natalizumab	4 (4%)
MRI activity in the previous year, number (%)	70 (69.3%)
Naïve, number (%)	51 (50.5%)
Neda loss at follow‐up, number (%)	23 (22.8%)
EDSS	
Baseline, median (range)	2 (0–6.5)
Follow‐up, median (range)	2.5 (0–6.5)
HADS‐Total, mean (SD)	12.4 (±6.35)
HADS‐A, mean (SD)	7.05 (±3.8)
HADS‐D, mean (SD)	5.35 (±3.5)
Performed under the cut‐off in ≥1 BICAMS subtest at baseline, number (%)	18 (17.8%)
Performed under the cut‐off in ≥2 BICAMS subtests at baseline, number (%)	6 (5.9%)

Abbreviations: BVMT‐R, brief visuospatial memory test, revised; CVLT‐II, California verbal learning test, 2nd edition; EDSS, Expanded Disability Status Scale; HADS, Hospital Anxiety and Depression Scale; PPMS, primary progressive multiple sclerosis; RRMS, relapsing–remitting multiple sclerosis; SD, standard deviation; SDMT, symbol digit modalities test; SPMS, secondary progression multiple sclerosis.

The number of patients classified as declined or improved at follow‐up using 80% and 90% of confidence levels for RCI and RB‐CI are reported in Table 2. Considering a 90% of confidence levels for RB‐CI, 12 (11.9%) improved at SDMT, 13 (12.9%) at CVLT‐II, and 13 (12.9%) at BVMT‐R; while 7 (6.9%) were classified as worsened at SDMT, 11 (10.9%) at CVLT‐II and 8 (7.9%) at BVMT‐R. During follow‐up, 23 (22.7%) patients lost NEDA‐3, including 17 (73%) patients that showed Progressive Independent of Relapse Activity (PIRA) events [28, 29].

**TABLE 2:** Neuropsychological changes performance at follow‐up using reliable cognitive change index (RCI) and regression‐based change index (RB‐CI) methodology.

		RC		RB‐CI	
		80%	90%	80%	90%
SDMT	Declined, number (%)	7 (6.9%)	6 (5.9%)	9 (8.9%)	7 (6.9%)
	Improved, number (%)	12 (11.9%)	8 (7.9%)	23 (22.8%)	12 (11.9%)
CVLT‐II	Declined, number (%)	12 (11.9%)	7 (6.9%)	14 (13.9%)	11 (10.9%)
	Improved, number (%)	25 (24.8%)	16 (15.8%)	21 (20.8%)	13 (12.9%)
BVMT‐R	Declined, number (%)	14 (13.9%)	7 (6.9%)	12 (11.9%)	8 (7.9%)
	Improved, number (%)	19 (18.8%)	11 (10.9%)	20 (19.8%)	13 (12.9%)

Abbreviations: BVMT‐R, brief visuospatial memory test, revised; CVLT‐II, California verbal learning test, 2nd edition; SDMT, symbol digit modalities test.

SDMT‐T scores significantly improved at follow‐up for the entire group (*p* = 0.003) and in patients maintaining NEDA‐3 (*p* < 0.001) (Table 3). At repeated‐measures ANCOVA, subjects with preserved NEDA‐3 status (*n* = 78) showed a higher increase in SDMT scores (+2.63 vs. −1.04 respectively), interaction time*NEDA‐3 status (*p* = 0.05) (data not shown).

**TABLE 3:** Mean scores on BICAMS subtests at baseline and after 24 months.

	Baseline		Follow‐up		*p*‐value
	Mean	SD	Mean	SD	
A. Global population					
SDMT (T‐score)	48.37	12.12	51.23	13.12	**0.003**
CVLT‐II (T‐score)	48.51	10.71	50.42	12.32	0.106
BVMT‐R (T‐score)	53.44	9.62	54.59	11.37	0.409
B. NEDA‐3 preserved, *N* = 78 (77.3%)					
SDMT (T‐score)	49.99	11.13	53.85	12.09	**<0.001**
CVLT‐II (T‐score)	49.38	9.79	51.65	11.72	0.096
BVMT‐R (T‐score)	54.48	9.59	54.91	11.75	0.774
C. NEDA‐3 loss, *N* = 23 (22.7%)					
SDMT (T‐score)	42.85	13.89	42.67	12.79	0.831
CVLT‐II (T‐score)	45.56	13.21	46.23	13.62	0.780
BVMT‐R (T‐score)	50.37	9.21	53.49	10.16	0.156

*Note*: Statistically significant *p*‐values (*p* < 0.05) are reported in bold; NEDA‐3, No evidence of disease activity defined as the absence of relapses, Expanded Disability Status Scale (EDSS) progression, and radiological activity.

Abbreviations: BVMT‐R, brief visuospatial memory test, revised; CVLT‐II, California verbal learning test, 2nd edition; SD, standard deviation; SDMT, symbol digit modalities test.

The multivariable regression models showed no significant results for CVLT‐II and BVMT‐R changes at follow‐up (data not shown). The model assessing the SDMT improvement (*n* = 12; *z* = 1.65), was significant and explained 21% of the variance (*p* = 0.038; Nagelkerke *R* ^2^ = 0.212). Lower EDSS proved to be an independent predictor of SDMT reliable improvement (*p* = 0.027) in our sample (See Table 4). The same regression model considering SDMT decline, was not significant (data not shown).

**TABLE 4:** Binary Logistic multivariable regression analysis exploring the association between demographic and clinical characteristics and SDMT improvements (RB‐CI 90%).

	*B*	95% CI		SE	*Z*	*p*
		Lower	Upper			
Disease duration	−0.0424	−0.1322	0.0473	0.0458	−0.927	0.354
EDSS at baseline	−0.7308	−13.769	−0.0847	0.3296	−2217	**0.027**
HADS‐total	0.0728	−0.0207	0.1663	0.0477	1526	0.127
MRI activity	0.2063	−12.245	16.371	0.7300	0.283	0.777
Previous treatment	1.0014	−0.4370	24.397	0.7339	1365	0.172

*Note*: Statistically significant *p*‐values (*p* < 0.05) are reported in bold.

Abbreviations: EDSS, Expanded Disability Status Scale; HADS, Hospital Anxiety and Depression Scale.

## DISCUSSION

Although cognitive impairment is common and disabling in MS, data on its long‐term progression in relation to treatment response are limited. Our study utilized the BICAMS battery to assess neuropsychological performance changes in a cohort of MS patients starting high‐efficacy treatment and investigated the effect of good disease control over cognitive trajectories. At baseline, 17.8% of our sample failed at least 1 test contrasting with previous reports citing prevalence rates between 32% and 70% in MS patients [4, 27, 30, 32]. This discrepancy might be due to the relatively small sample size and the different population characteristics of the previously mentioned studies. It is also noteworthy to remember that many studies on cognitive impairment in MS were conducted before the introduction of most “high‐efficacy” DMTs. Our cohort had a mean disease duration of <10 years, median baseline EDSS of 2, and 69.3% showed MRI activity in the previous year. Around 50% were treatment‐naïve at baseline, all requiring “high efficacy” DMT initiation.

The low proportion of impairment detected in our recently assessed population—together with the highly variable incidence rate reported in previous studies—highlights the importance of carrying out larger, continuous, and updated analyses assessing cognitive impairment in the context of the recent treatments' scenario.

Assessing changes in neuropsychological assessments is a complex matter since it requires accounting for random fluctuations and learning effect [9]. Recently, Portaccio et al. [13], provided a RB‐CI calculator to estimate a “normalized” and individualized change in cognitive performance at various neuropsychological tests. As reported by the authors, the use of a regression‐based change index allows to consider practice effect, age, sex, education, and baseline score on an individualized basis, in the cognitive change estimation. The advantage of RB‐CI seems to be particularly relevant for cognitive improvement since it does not use the same cut‐off for both declining and improving performance. Given that this calculation does not consider common MS comorbidities, such as depression and anxiety, the total HADS score was considered in our regression analysis, together with disease duration, EDSS, MRI activity, and previous treatment. Additionally, to further reduce the practice effect, our patients performed a single retest after 2 years interval with parallel versions of the battery [8, 25, 27, 31, 32].

In our sample, 12 (11.9%) MS patients showed SDMT improvement using the conservative 90% RB‐CI confidence interval, therefore, our study seems to support the possibility of cognitive performance improvement in patients undergoing high‐efficacy therapy [17].

Patients who maintained NEDA‐3 status during follow‐up showed greater SDMT improvement suggesting that effective disease control could favour a positive effect of DMTs on cognition [16, 33]. These findings are in line with recent evidence showing that early commencement of highly efficacy drugs is associated with reduced long‐term physical and cognitive disability [14, 16, 34, 35].

In the final phase of our study, we aimed to investigate the association between demographic and clinical characteristics and cognitive changes, seeking to identify variables that could predict cognitive performance decline or improvement over the follow‐up period. Our analysis showed that lower EDSS independently predicts SDMT improvement at follow‐up, highlighting that a premature intervention, when disability is still low, could positively influence not only physical but also cognitive performance. Accordingly, we believe that cognitive assessment could be fully integrated into the concept of disease progression, as previously suggested by various authors [6, 16]. Even though the SDMT score was the only measure to show significant improvement from baseline to follow‐up, the use of the entirely BICAMS allowed us to identify approximately 4%–5% more cognitively impaired patients compared to SDMT alone. Therefore, we recommend using the entire BICAMS battery as a tool for identifying cognitively compromised patients.

Our study is not without limitations. Cognitive improvement in MS appears to be variable, and using a larger number of tests could provide more comprehensive information. Among the common comorbidities in MS, fatigue might affect also neuropsychological performance [36]; unfortunately, given the clinical nature of our study, we were not able to assess this aspect, which should be taken into account in future analyses. Additionally, due to a relatively small sample size, only a very small number of patients showed a reliable cognitive decline during follow‐up, thus limiting the power of predictive analyses. Further studies with larger sample sizes and more comprehensive evaluations should be considered to assess these important matters.

To conclude, our findings showed that early disease activity control—especially in patients with low baseline disability—may yield significant benefits even in terms of cognitive performance.

## AUTHOR CONTRIBUTIONS

**E. Leveraro:** Conceptualization; writing – original draft; writing – review and editing; investigation; data curation; formal analysis. **M. Cellerino:** Conceptualization; writing – review and editing; formal analysis; writing – original draft. **C. Lapucci:** Supervision. **M. Dighero:** Data curation. **L. Nasone:** Writing – review and editing. **T. Sirito:** Writing – review and editing. **D. Boccia:** Writing – review and editing; formal analysis. **N. Cavalli:** Writing – review and editing. **G. Bavestrello:** Writing – review and editing. **A. Uccelli:** Supervision. **G. Boffa:** Formal analysis. **M. Inglese:** Supervision; project administration.

## CONFLICT OF INTEREST STATEMENT

Dr. M. Inglese received grants NIH, NMSS, FISM; received fees for consultation from BMS; Janssen, Roche, Genzyme, Merck, Biogen, and Novartis. Dr. M. Cellerino received personal compensations from Novartis, Sanofi Genzyme, Teva, and consulting fees from Zambon. Dr. G. Boffa was supported by a research fellowship FISM—Fondazione Italiana Sclerosi Multipla 2019/BR/016 and financed or co‐financed with the “5 per mille” public funding. He received personal fee from Novartis and Roche. Dr. C. Lapucci received personal fee from Novartis, Roche, Sanofi, BMS, and Janssen. Dr. Uccelli received grants from FISM, Biogen, Roche, Alexion, and Merck Serono. All other authors declare no competing interests.

## Data Availability

The data presented in this study are available on request from the corresponding author.
